# Association study in siblings and case-controls of serotonin- and oxytocin-related genes with high functioning autism

**DOI:** 10.1186/2049-9256-2-1

**Published:** 2014-01-24

**Authors:** Johanna Nyffeler, Susanne Walitza, Elise Bobrowski, Ronnie Gundelfinger, Edna Grünblatt

**Affiliations:** University Clinics of Child and Adolescent Psychiatry (UCCAP), University of Zurich, Thurgauerstr. 39, CH-8050 Zurich, Switzerland; Neuroscience Center Zurich, University of Zurich and ETH Zurich, Zurich, Switzerland; Department of Experimental Psychology, University of Regensburg, Regensburg, Germany

**Keywords:** Autism spectrum disorder, High functioning autism, Oxytocin receptor, Polymorphism, Serotonin receptor 2A, Serotonin transporter

## Abstract

**Background:**

Autism spectrum disorder (ASD) is heritable and neurodevelopmental with unknown causes. The serotonergic and oxytocinergic systems are of interest in autism for several reasons: (i) Both systems are implicated in social behavior, and abnormal levels of serotonin and oxytocin have been found in people with ASD; (ii) treatment with selective serotonin reuptake inhibitors and oxytocin can yield improvements; and (iii) previous association studies have linked the serotonin transporter (*SERT*; *SLC6A4*), serotonin receptor 2A (*HTR2A*), and oxytocin receptor (*OXTR*) genes with ASD. We examined their association with high functioning autism (HFA) including siblings and their interaction.

**Methods:**

In this association study with HFA children (IQ > 80), siblings, and controls, participants were genotyped for four single nucleotide polymorphisms (SNPs) in *OXTR* (rs2301261, rs53576, rs2254298, rs2268494) and one in *HTR2A* (rs6311) as well as the triallelic HTTLPR (SERT polymorphism).

**Results:**

We identified a nominal significant association with HFA for the HTTLPR s allele (consisting of S and L_G_ alleles) (p = .040; odds ratio (OR) = 1.697, 95% CI 1.191–2.204)). Four polymorphisms (HTTLPR, *HTR2A* rs6311, *OXTR* rs2254298 and rs53576) in combination conferred nominal significant risk for HFA with a genetic score of ≥4 (OR = 2.09, 95% CI 1.05–4.18, p = .037). The resulting area under the receiver operating characteristic curve was 0.595 (p = .033).

**Conclusions:**

Our findings, combined with those of previous reports, indicate that ASD, in particular HFA, is polygenetic rather than monogenetic and involves the serotonergic and oxytocin pathways, probably in combination with other factors.

**Electronic supplementary material:**

The online version of this article (doi:10.1186/2049-9256-2-1) contains supplementary material, which is available to authorized users.

## Background

Autism spectrum disorder (ASD) is a neurodevelopmental disorder characterized by impairments in social interactions and communication and by repetitive behaviors [1, 2]. An ASD diagnosis can be made very early in childhood, but the disorder is a lifelong condition. The prevalence is estimated to be 0.6–1.0% [3–6], with a male:female ratio of 4:1 [6]. Twin studies give an estimated heritability of 70–90% [7–10], implicating genetics as a main factor in the etiology, in addition to environmental factors. Some ASD cases are caused by single gene defects [11–13], but for most cases, the genetic causes are unknown.

Both the serotonergic and oxytocinergic systems seem to play a role in ASD and social behaviors [14]. The serotonergic system is of special interest in autism for several reasons: (i) In up to 30% of people with ASD, elevated whole blood serotonin (5-HT) levels have been reported [15]; (ii) ASD-related sensory motor behaviors are increased after depletion of tryptophan, a precursor in 5-HT synthesis [16]; (iii) reduced serotonin receptor 2A (HTR2A) and serotonin transporter (SERT, also known as 5-HTT) binding in certain brain regions of people with ASD has been identified [17–19]; (iv) selective serotonin reuptake inhibitors (SSRIs) can improve abnormal reciprocal social interaction and repetitive behaviors in some cases [20–23]; and (v) several polymorphisms in the *HTR2A* gene (NCBI Gene ID: 3356) and the *SERT* gene (also known as *SLC6A4*; NCBI Gene ID: 6532) have suggested association with ASD [24–29]. In the following, we focus on these two candidate genes: The promoter sequence of *SERT* contains a polymorphic region (HTTLPR) with a short allele (S) and a long allele (L) that is 44 bp longer and can contain an additional single nucleotide polymorphism (SNP) (rs25531), making the locus triallelic (L_A_, L_G_ and S) [30, 31]. These polymorphisms affect *SERT* expression, with the S and L_G_ alleles (here denoted collectively as the s allele, while L_A_ as the l allele) reducing transcriptional efficiency [30, 31]. Still, some inconsistent results appear with s allele or l allele associations with ASD depending on ethnicity or diagnostic inclusion as found in a meta-analysis [24]. Association studies of *HTR2A* have concentrated mostly on three non-coding SNPs (rs6311, rs6313, rs6314) [26–28, 32].

Similarly, the oxytocinergic system has attracted attention for similar reasons: (i) Low plasma oxytocin levels have been observed in autistic boys [33, 34]; (ii) elevated oxytocin precursor levels in ASD children have been reported [33]; and (iii) administration of oxytocin has improved retention of social information and decreased repetitive behaviors in ASD as well as in high functioning autism (HFA) [35–38]. Nonetheless, genetic studies have mainly failed to associate the *oxytocin* gene with autism; however, several studies have reported an association with the oxytocin receptor gene (*OXTR*; NCBI Gene ID: 5021), although inconsistently [39–43]. In particular the *OXTR* polymorphism: rs2301261, rs53576, rs2254298 and rs2268494, were studied in ASD and social behavior [40, 42–44].

Recent reports have indicated some interactions among 5-HT, serotonergic components, and oxytocin [14, 45, 46]. In the first report by Hammock et al. [45], plasma oxytocin and 5-HT levels were negatively correlated with each other, and this relationship was most prominent in children under age 11 years. Thanseem and colleagues [46], on the other hand, found that transcription factor–like specificity protein 1 expression in brains of ASD participants increased in parallel with dysregulation of the transcription of *HTR2A* (down-regulation) and *OXTR* (up-regulation), which might further reveal downstream pathways mediating brain developmental disorders. Moreover, Dolen et al. [14] could demonstrate in mice models that the rewarding properties of social interactions require the coordinated activity of oxytocin and 5-HT in the nucleus accumbens, and this oxytocin-induced synaptic plasticity requires activation of nucleus accumbens serotonin receptor 1B.

Because previous association studies of *SERT*, *HTR2A*, and *OXTR* have led to controversial findings, but the mentioned genes seem to interact with one another, we attempted to replicate these associations analyzing ASD children (high functioning), their siblings, and controls with no clinical diagnoses. In contrast to other studies, we included only patients with HFA in our study, eliminating confounding parameters such as IQ. Sibships transmission analysis was included to enhance further the case–control findings. Additionally, we tested for interaction among the three genes, since these were reported to be involved in ASD and even further to interact with each other, on the assumption that neurodevelopmental disorders are polygenetic rather than monogenetic.

## Methods

### Participants

The study was approved by the ethics committee of the Canton Zurich, Switzerland (E-36/2009). Parents of all participants gave their written consent after being informed about the aim of the study. All participants (76 with HFA, 78 siblings, and 99 controls) were Caucasians between 5 and 17 years of age collected in the Department of Child and Adolescent Psychiatry at the University of Zurich. In all patients diagnosis was confirmed using the Autism Diagnosis Observation Schedule [47] and the Autism Diagnosis Interview [48]. Inclusion criteria for all children with high-functioning ASD (64 males and 12 females) was IQ of at least 80 from at least one of two IQ tests (see below) according to strict HFA definition [49, 50] and all met the International Statistical Classification of Diseases and Related Health Problems, 10th Revision (ICD-10) [51] criteria for pervasive developmental disorder, including three with childhood autism, 27 with atypical autism, and 46 with Asperger syndrome. Persons with neurological disorders including epilepsy or known genetic diseases linked to autism were excluded.

The siblings of the ASD group (33 males and 45 females) did not have an ASD diagnosis or other severe psychiatric disorders according to screening questionnaires (see below). For the control group, only children without any clinical diagnosis were included in the study (77 males and 22 females).

Additionally, all participants were screened for psychopathology with the following parent reports: Child Behaviour Checklist [52]; Social Responsiveness Scale [53]; Social Communication Questionnaire [54]; Conners [55]; and the German ADHD rating scale, FBB-HKS [56]. Intelligence was measured with the Snijders-Oomen Non-Verbal Intelligence Test 5.5-17 [57] and the Culture Fair Test [58]. Age and IQ distribution for the different study groups is listed in Table 1.

**Table 1:** **Distribution of age, sex, and IQ for each study group**

		Mean	SD	Range	Total
Age (in y)	ASD	11.24	3.10	5–17	76
	Siblings*	10.53	3.25	6–17	78
	Controls	11.77	3.01	6–17	99
IQ, SON	ASD	108.68	16.03	80–140	75
	Siblings	109.75	12.36	82–135	77
	Controls	112.19	12.53	86–140	99
IQ, CFT	ASD	105.16	13.88	70–145	68
	Siblings	104.55	10.65	85–142	73
	Controls	106.71	10.96	85–133	97
		**Male**	**Female**	**Ratio (M/F)**	**Total**
		**(% total)**	**(% total)**		
Sex	ASD	64 (84.2)	12 (15.8)	5.33	76
	Siblings^*+^	33 (42.3)	45 (57.7)	0.733	78
	Controls	77 (77.8)	22 (22.2)	3.50	99

*Abbreviations*: *ASD* autism spectrum disorder, *SD* standard deviation, *SON* Snijders-Oomen Non-Verbal Intelligence Test 5.5-17, *CFT* Culture Fair Test, *M* Male, *F* Female.

Statistical analysis was conducted using χ^2^ tests. *p < .05 versus controls; ^+^p < .05 versus ASD.

### Genotyping analysis

Saliva samples for DNA isolation were collected from all recruited individuals using the Oragene DNA kit (DNA Genotek; Kanata, Canada). DNA was isolated from saliva according to the manufacturer’s protocol (Oragene™ DNA Purification Protocol, DNA Genotek). For rs2301261, rs2254298, rs2268494, and rs6311 (Assay ID: C_15756091_30; C_15981334_10; C_15874471_10 and C_8695278_10, respectively), genotyping was performed using TaqMan® SNP Genotyping Assays (Applied Biosystems; Foster City, CA, USA). PCR was carried out on a CFX384™ Real-Time System (Bio-Rad; Hercules, CA, USA) in a 5 μl (10 μl for rs6311) volume using TaqMan® 2× Universal PCR Master Mix No AmpErase® UNG (Applied Biosystems) and 10 ng (22.5 ng for rs6311) of DNA. Initial enzyme activation was carried out at 95°C for 10 min, followed by 40 cycles at 92°C for 15 s and 60°C for 1 min.

For rs25531 and HTTLPR analysis, the restriction fragment length polymorphism method was used. Amplification was carried out on a CFX384™ Thermal Cycler (Bio-Rad) in a 10 μl (rs25531) or 25 μl (HTTLPR) volume using GoTaq® Green Master Mix 2× (Promega; Madison, WI, USA).

For rs25531, the same primers as described previously were used [59]. PCR conditions were an initial denaturation at 95°C for 2 min, followed by 40 cycles at 95°C for 30 s, 64°C for 40 s, and 72°C for 40 s with a final extension at 72°C for 5 min. The PCR product was digested overnight at 37°C with 10 U BamHI (Fermentas; Burlington, Canada) in a 20 μl volume containing 2 μl of the corresponding enzyme buffer. Fragments were visualized on a 3% agarose gel. The fragment size of the undigested G allele is 340 bp whereas the A allele is restricted to bands of 110 and 230 bp.

For HTTLPR, the primer sequences were 5′-TGC CGC TCT GAA TGC CAG CAC-3′ and 5′-GGG ATT CTG GTG CCA CCT AGA CG-3′. PCR conditions were similar to those for rs53576, but only 30 cycles were carried out at 95°C for 45 s, 66.5°C for 45 s, and 72°C at 1 min. Fifteen microliters of PCR product were run on a 3% agarose gel to distinguish the L allele (463 bp) and S allele (419 bp). The remaining PCR product was digested similarly as described above but with 20 U MspI (New England Biolabs, Ipswich, MA, USA). Visualization on 3% agarose gel allowed distinction of the G allele (bands of 61, 66, 162, 174, and 292 bp) from the A allele (61, 66, 292, and 336 bp) of rs25531.

### Statistical analysis

Each study group and the total sample were tested for deviation from Hardy–Weinberg equilibrium for all polymorphisms, and no significant departures were found (see Additional file 1: Table S1). Differences in genotype, allele, and carrier frequencies among the groups (HFA, controls, siblings) as well as between two HFA subgroups (atypical autism and Asperger autism) and the control group were tested with the χ^2^ test. A sibship disequilibrium test was performed for each polymorphism according to Horvath and Laird [60].

Gene–gene interactions were studied by comparing each combination of two polymorphisms. For each combination, a three-dimensional contingency table with “polymorphism 1 × polymorphism 2 × study group” was built, and a three dimensional χ^2^ test was performed. All analyses were done with Matlab version 7.10.0 (MathWorks).

Additionally, a receiver operating characteristic (ROC) curve analysis was performed for the various combinations of genetic scores sums, simulating their polygenetic effects on the risk for ASD [61]. Genetic scores attributed to the allelic variations are listed in Additional file 1: Table S2. The area under the curve and its significance were calculated using SPSS version 20 (IBM Corp.).

The nominal significance threshold was set to 5% and the adjusted significance according Bonferroni for multiple testing was set to 0.8%. Power analysis was performed using G*Power version 3.1.6 [62, 63].

## Results

### Association of single SNPs with autism diagnosis

To investigate whether oxytocinergic and serotonergic system genes are associated with HFA, 253 children were genotyped for polymorphisms in the *OXTR*, *HTR2A*, and *SERT*. Genotype and minor allele frequencies for all six polymorphisms are summarized in Table 2.

**Table 2:** **Genotype distribution, genotype frequencies, and minor allele frequencies for autism spectrum disorder (ASD), siblings, and control groups**

Polymorphism	Genotype distribution (Genotype frequency)			MAF (Allele)
**HTTLPR triallelic** ^**1**^	**ss**	**sl**	**ll**	
ASD	24 (0.316)	38 (0.500)	14 (0.184)	0.434 (l)
Siblings	16 (0.208)	43 (0.558)	18 (0.234)	0.487 (s)
Controls	22 (0.248)	51 (0.526)	24 (0.247)	0.490 (s)
***HTR2A*** **rs6311** ^**2**^	**GG**	**GA**	**AA**	**A**
ASD	21 (0.276)	34 (0.447)	21 (0.276)	0.500
Siblings	23 (0.295)	35 (0.449)	20 (0.256)	0.481
Controls	29 (0.299)	47 (0.485)	21 (0.216)	0.459
***OXTR*** **rs2301261**	**CC**	**CT**	**TT**	**T**
ASD	65 (0.855)	11 (0.145)	0	0.072
Siblings	67 (0.859)	11 (0.141)	0	0.071
Controls	84 (0.848)	15 (0.152)	0	0.076
***OXTR*** **rs53576** ^**1**^	**AA**	**AG**	**GG**	**A**
ASD	7 (0.092)	34 (0.447)	35 (0.461)	0.316
Siblings	7 (0.091)	37 (0.481)	33 (0.429)	0.331
Controls	9 (0.093)	33 (0.340)	55 (0.567)	0.263
***OXTR*** **rs2254298**	**AA**	**AG**	**GG**	**A**
ASD	0	12 (0.158)	64 (0.842)	0.079
Siblings	0	13 (0.167)	65 (0.833)	0.083
Controls	0	21 (0.212)	78 (0.788)	0.106
***OXTR*** **rs2268494**	**AA**	**AT**	**TT**	**A**
ASD	0	13 (0.171)	63 (0.829)	0.086
Siblings	1 (0.013)	9 (0.115)	68 (0.872)	0.071
Controls	0	17 (0.172)	82 (0.828)	0.086

*Abbreviations*: *ASD* autism spectrum disorder, *OXTR* oxytocin receptor, *HTR2A* serotonin receptor 2A, *HTTLPR* serotonin-transporter–linked polymorphic region, *MAF* minor allele frequency. ^1^Genotyping failed repeatedly for one sibling and two controls. ^2^Genotyping failed repeatedly for two controls.

HTTLPR triallelic polymorphism allele frequencies were nominal significantly associated with Asperger diagnosis (p = .040; odds ratio (OR) = 1.697 (95% CI 1.191–2.204)) with the s allele as a risk allele (Table 3). The absolute genotype frequencies for the Asperger group were 17, 23, and 6 for ss, sl, and ll genotypes, respectively, for a relative s allele frequency of 0.619 for the Asperger group compared to 0.490 in the control group. Furthermore, merging l carriers led to a trend for association (p = .073; OR = 1.998 (95% CI 1.234–2.763)). No association of the *HTR2A* and *OXTR* with autism was observed.

**Table 3:** **Statistical association analysis between autism spectrum disorder (ASD) or its subgroups (atypical and Asperger) compared to controls or siblings with all six polymorphisms**

	p values			
Polymorphism	AA vs. controls	AS vs. controls	ASD vs. controls	ASD vs. siblings
**Genotype frequencies**				
HTTLPR triallelic	0.926	0.110	0.350	0.301
*HTR2A* rs6311	0.720	0.299	0.661	0.949
*OXTR* rs2301261	0.595	0.731	0.901	0.948
*OXTR* rs53576	0.524	0.280	0.334	0.914
*OXTR* rs2254298	0.460	0.592	0.363	0.883
*OXTR* rs2268494	0.870	0.974	0.991	0.388
**Allele frequencies**				
HTTLPR triallelic	0.708	**0.040**	0.160	0.168
*HTR2A* rs6311	0.591	0.410	0.446	0.736
*OXTR* rs2301261	0.609	0.743	0.905	0.950
*OXTR* rs53576	0.307	0.464	0.280	0.774
*OXTR* rs2254298	0.486	0.614	0.390	0.888
*OXTR* rs2268494	0.877	0.975	0.991	0.623
**Carrier frequencies [Carrier]**				
HTTLPR triallelic [s]	0.787	0.108	0.319	0.451
HTTLPR triallelic [l]	0.725	0.073	0.189	0.129
*HTR2A* rs6311 [C]	0.949	0.158	0.362	0.780
*HTR2A* rs6311 [T]	0.433	0.948	0.744	0.799
*OXTR* rs53576 [G]	0.776	0.579	0.988	0.980
*OXTR* rs53576 [A]	0.258	0.216	0.164	0.691

In the subgroup analysis the three childhood autism were not included in either AA or AS subgroup. *Abbreviations*: *AA* atypical autism, *AS* Asperger syndrome, *ASD* autism spectrum disorder, *OXTR* oxytocin receptor, *HTR2A* serotonin receptor 2A, *HTTLPR* serotonin-transporter–linked polymorphic region, *vs.* versus, ***Bold*** nominal significant.

Forty “mixed” sibships were included (sibships with affected and unaffected siblings). No significant transmission disequilibrium was detected for any of the tested polymorphisms (Table 4).

**Table 4:** **Results of the sibship disequilibrium test**

Polymorphism	Allele	b	c	p
HTTLPR triallelic	l	8	12	.503
*HTR2A* rs6311	A	6	10	.455
*OXTR* rs2301261	C	5	1	.219
*OXTR* rs53576	T	6	8	.791
*OXTR* rs2254298	G	1	2	1.000
*OXTR* rs2268494	T	2	4	.688

*Abbreviations*: *OXTR* oxytocin receptor, *HTR2A* serotonin receptor 2A, *HTTLPR* serotonin-transporter–linked polymorphic region. b = mentioned allele more often transmitted to affected siblings. c = mentioned allele more often transmitted to unaffected siblings. p = two-tailed p value.

### Analysis of gene–gene interactions and the polygenetic risk for ASD

Testing for gene–gene interactions by a χ^2^-test revealed nominal significant association of HTTLPR and *HTR2A* (rs6311) with ASD, as well as of the two *OXTR* SNPs rs53576 and rs2268494 (Additional file 1: Table S3). Three SNPs on the *OXTR* showed strong linkage disequilibrium; between rs2301261 and rs2254298 (p = 4.58E-11) and between rs2254298 and rs2268494 (p = .005); probably causing high transmission of these variant combinations.

**Figure 1:**
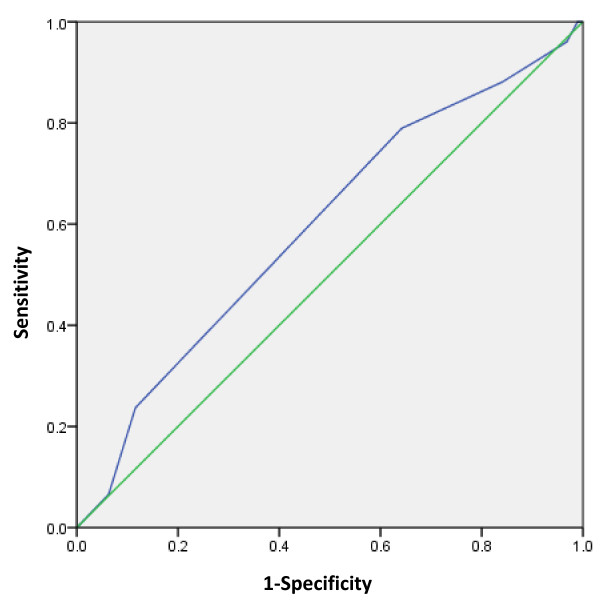
**Receiver operating characteristic curve for the candidate polymorphism markers, serotonin transporter promoter length polymorphism (HTTLPR), serotonin receptor 2A (** ***HTR2A*** **) rs6311, oxytocin receptor (** ***OXTR*** **) rs2254298, and** ***OXTR*** **rs53576.** AUC = 0.595, p = .033, 95% CI: 0.509–0.681. Sensitivity of ~80% with specificity of ~35% or sensitivity of ~35% with specificity of ~80%.

Since there is evidence in the literature for the involvement of oxytocinergic and serotonergic systems and their interactions with one another, we investigated the polygenetic gene scores of these variants and the risk for HFA. The strongest result for a polygenetic risk was with the combination of the polymorphisms HTTLPR, *HTR2A* rs6311, and *OXTR* rs2254298 and rs53576 (for other combinations, see Additional file 1: Table S4). A nominally significant ROC curve (p = .033) was obtained for this combination (Figure 1). The optimal point is at ~80% sensitivity and ~35% specificity or ~35% sensitivity with ~80% specificity. A genetic score cutoff of 4 or more, indicating more risk variants, resulted in an OR = 2.090 (95% CI 1.045–4.179, p = .037).

## Discussion

ASD is a highly heritable neurodevelopmental disorder with suggested involvement of the serotonergic and oxytocinergic systems, but up to now, no clear association of polymorphisms with ASD has been found with a high effect size. Here, we evaluated children with HFA, their siblings, and controls using genotyping results for four SNPs in *OXTR*, one in *HTR2A*, and the HTTLPR length polymorphism. Our analysis showed that the s allele of the HTTLPR polymorphism was nominal significantly associated with HFA; however, it should be denoted that due to sample size the p-value is rather borderline. We did not observe any significant association in the selected SNPs for the *HTR2A* and *OXTR* variants with HFA.

Regarding association with *HTR2A*, our findings are consistent with some previous studies [26, 28, 32] reporting no association with autism. Only one study involving patients “rather severely affected from autism” [27] reported a link, but no information regarding IQ values was given.

Association studies of HTTLPR with ASD have led to contradictory results. Several have identified the S allele with ASD [25, 29, 64, 65] while others associated the L allele with ASD [32, 66, 67] or found no association at all [68–71]. Our results rather support the first group. In our analysis of the HTTLPR, however, we used a triallelic mode in which the A and G alleles in the L allele were taken into account.

In accordance with previous reports [42, 72] and a recent meta-analysis finding [44] in which no association between the *OXTR* polymorphisms rs53576 and rs2301261 with ASD was found, we also could not confirm one with HFA. Lerer et al. [40] identified an association between *OXTR* rs2268494 and autism diagnosis, but only when IQ was entered as a covariate. In our study, although our ASD population was stratified to IQ equal or larger than eighty, we could not confirm such a link. Concerning *OXTR* rs2254298, we also could not confirm an association with HFA, in contrast to several previous studies [40, 42, 59, 73]. Nevertheless, our finding supports the recent meta-analysis in which no association could be proven accept for the biological functioning domain [44].

Although only one SNP singly was associated with HFA in this work, the gene–gene interaction study linked combinations of the tested polymorphisms with HFA. Similarly, such gene interaction study between *HTTLPR* and *OXTR* was reported in prediction of maternal sensitivity [74], pointing to their possible influence in social behavior. Furthermore, the ROC analysis showed that four of the tested SNPs together led to sensitivity of 80% but at the cost of low specificity (35%) or vice versa.

The limitations of our study include the relatively small minor allele frequency for three of the *OXTR* polymorphisms and the sample size. Power analysis revealed that the power was sufficient only for a medium or large effect size. The power for small effect sizes (i.e., of 0.1) was below 20% for polymorphisms with two genotypes and about 30% if all three genotypes were present.

The strength of our study is the narrow phenotype regarding the intellectual and language ability and cognitive function. Most previous investigations have analyzed samples consisting of the complete spectrum of autism whereas in our investigation all individuals were diagnosed with HFA. In the past, 50–70% of autistic children were classified as intellectually disabled, but those with Asperger have, by definition, an IQ in the normal range [75] and typical language development.

As far as we know, only one study has investigated an association of *OXTR* in HFA, finding a weak association [72]. Of the 22 studied SNPs, one was nominally associated with autism diagnosis (p = .0185), which would not hold for Bonferroni correction for multiple testing [72]. Further research involving people with HFA has yielded evidence for an association with the *oxytocin* gene itself [76]; the *CD38* gene [77], whose gene product is related to oxytocin secretion [78–80]; and the syntaxin 1A gene [81], whose gene product affects SERT function [82]. These findings indicate that some components of the serotonergic and oxytocinergic systems, other than those already extensively studied, might be involved in HFA. Additionally, similarly to the report by Carayol et al. [61], who combined the low-risk genes *PITX*, *ATP2B2*, *SLC25A12*, and *EN2*, we could show that a combination of four polymorphisms (HTTLPR, *HTR2A* rs6311, and *OXTR* rs2254298 and rs53576) confers a nominal significant risk for HFA. This result points to the possibility that these genes play a role in ASD, probably in combination with additional risk genes that should be further explored.

## Conclusions

In summary, many studies have found associations of *OXTR*, *HTR2A*, and *SERT* with ASD, but we could not confirm these with HFA except for a nominal association with the HTTLPR polymorphism. Our findings might be explained by the fact that HFA individuals have different symptoms from others with ASD and by the wide heterogeneity in the ASD population. Of interest, however, a combination of those polymorphisms resulted in nominal significant risk for HFA, pointing to the importance of a polygenetic rather than monogenetic context, in which each gene contributes to a very small fraction of the phenotype. Therefore, we suggest that future association studies should look into this aspect and examine various combinations of risk genes with HFA.

## Availability of supporting data

The data sets supporting the results of this article are included within the article (and its additional files).

## Electronic supplementary material

Additional file 1: Table S1: Results of testing for deviation from Hardy–Weinberg equilibrium. **Table S2.** Allelic variation used in the calculation of genetic score under an additive model. **Table S3.** Gene–gene interactions and their associations with autism spectrum disorder (ASD). **Table S4.** Receiver operating characteristic curve analysis results for the different combinations of polygenetic risk for autism spectrum disorder (ASD). (DOC 152 KB)

## Abbreviations

ASD: Autism spectrum disorder
HFA: High functioning autism
5-HT: Serotonin
HTR2A: Serotonin receptor 2A
HTTLPR: Promoter sequence of *SERT* contains a polymorphic region
ICD-10: International statistical classification of diseases and related health problems, 10th Revision
OR: Odds ratio
OXTR: Oxytocin receptor
ROC: Receiver operating characteristic
SERTSLC6A4: Serotonin transporter
SNP: Single nucleotide polymorphism
SSRIs: Selective serotonin reuptake inhibitors.
