# Supplementary material for: Association study in siblings and case-controls of serotonin- and oxytocin-related genes with high functioning autism
Source: J Mol Psychiatry. 2014 Jan 24;2(1):1. doi: 10.1186/2049-9256-2-1 (PMC4223888; doi:10.1186/2049-9256-2-1)
Supplement: Supplementary file 1 — Additional file 1: Table S1: Results of testing for deviation from Hardy–Weinberg equilibrium. Table S2. Allelic variation used in the calculation of genetic score under an additive model. Table S3. Gene–gene interactions and their associations with autism spectrum disorder (ASD). Table S4. Receiver operating characteristic curve analysis results for the different combinations of polygenetic risk for autism spectrum disorder (ASD). (DOC 152 KB) [file 40303_2014_19_MOESM1_ESM.doc]

**Supplementary table S1**: Results of testing for deviation from Hardy–Weinberg equilibrium.

|  | **p value** | | | |
| --- | --- | --- | --- | --- |
| **Polymorphism** | **ASD** | **Siblings** | **Controls** | **All** |
| HTTLPR triallelic | .878 | .302 | .609 | .371 |
| *HTR2A* rs6311 | .359 | .371 | .811 | .242 |
| *OXTR* rs2301261 | .496 | .503 | .415 | .210 |
| *OXTR* rs53576 | .759 | .457 | .229 | .880 |
| *OXTR* rs2254298 | .455 | .422 | .238 | .112 |
| *OXTR* rs2268494 | .415 | .290 | .350 | .577 |

Abbreviations: ASD, autism spectrum disorder; OXTR, oxytocin receptor; HTR2A, serotonin receptor 2A; HTTLPR, serotonin-transporter–linked polymorphic region.

**Supplementary table S2:** Allelic variation used in the calculation of genetic score under an additive model.

| **Polymorphism** | **Risk allele** | **Genetic score = 0** | **Genetic score = 1** | **Genetic score = 2** |
| --- | --- | --- | --- | --- |
| HTTLPR | S | LL | SL | SS |
| *HTR2A* rs6311 | A | GG | AG | AA |
| *OXTR* rs2301261 * | C | CC/CT |  | CC |
| *OXTR* rs53576 | A | GG | AG | AA |
| *OXTR* rs2254298 * | G | AG/AA |  | GG |
| *OXTR* rs2268494 * | A | TT |  | AT/AA |

* Recessive transmission model

Annotations: HTTLPR, serotonin-transporter–linked polymorphic region; *HTR2A*, serotonin receptor 2A; *OXTR*, oxytocin receptor.

**Supplementary table S3:** Gene–gene interactions and their associations with autism spectrum disorder (ASD).

| **Gene 1** | **Gene 2** |  | **ASD vs. controls** | **ASD vs. siblings** | **All groups** |  | **LD** |
| --- | --- | --- | --- | --- | --- | --- | --- |
| HTTLPR triallelic | *HTR2A* rs6311 |  | **1.71E-02** | **2.53E-02** | **2.88E-02** |  | 5.40E-01 |
| HTTLPR triallelic | *OXTR* rs2301261 |  | 5.24E-02 | **1.85E-02** | 6.69E-02 |  | 6.41E-01 |
| HTTLPR triallelic | *OXTR* rs53576 |  | 9.12E-02 | 1.48E-01 | 1.20E-01 |  | 7.55E-01 |
| HTTLPR triallelic | *OXTR* rs2254298 |  | 1.41E-01 | 2.17E-01 | 2.20E-01 |  | 8.81E-01 |
| HTTLPR triallelic | *OXTR* rs2268494 |  | 2.11E-01 | 1.36E-01 | 2.08E-01 |  | 7.06E-01 |
| *HTR2A* rs6311 | *OXTR* rs2301261 |  | 5.70E-01 | 6.42E-01 | 7.32E-01 |  | 7.44E-01 |
| *HTR2A* rs6311 | *OXTR* rs53576 |  | 2.57E-01 | 7.26E-01 | 3.78E-01 |  | 7.92E-01 |
| *HTR2A* rs6311 | *OXTR* rs2254298 |  | 1.53E-01 | 4.41E-01 | 2.52E-01 |  | 4.01E-01 |
| *HTR2A* rs6311 | *OXTR* rs2268494 |  | 6.04E-02 | **1.22E-02** | **1.36E-02** |  | 5.73E-01 |
| *OXTR* rs2301261 | *OXTR* rs53576 |  | 6.58E-02 | **3.88E-02** | **1.57E-02** |  | 6.00E-02 |
| *OXTR* rs2301261 | *OXTR* rs2254298 |  | **1.38E-13** | **5.57E-03** | **7.09E-14** |  | **4.58E-11** |
| *OXTR* rs2301261 | *OXTR* rs2268494 |  | 2.33E-01 | 3.16E-01 | 2.27E-01 |  | 6.39E-01 |
| *OXTR* rs53576 | *OXTR* rs2254298 |  | 5.96E-02 | 1.13E-01 | **2.11E-02** |  | 1.33E-01 |
| *OXTR* rs53576 | *OXTR* rs2268494 |  | **4.14E-03** | **1.19E-02** | **6.28E-03** |  | 1.44E-01 |
| *OXTR* rs2254298 | *OXTR* rs2268494 |  | **2.15E-03** | **2.37E-02** | **2.81E-03** |  | **5.10E-03** |

Abbreviations: ASD, autism spectrum disorder; OXTR, oxytocin receptor; HTR2A, serotonin receptor 2A; HTTLPR, serotonin-transporter–linked polymorphic region; LD, linkage disequilibrium; **Bold**, nominal significant.

**Supplementary table S4:** Receiver operating characteristic curve analysis results for the different combinations of polygenetic risk for autism spectrum disorder (ASD).

| Polymorphism combination | AUC | SD | CI |  | Asymptotic  significance ^1^ |
| --- | --- | --- | --- | --- | --- |
|  |  |  | Low | High |  |
| HTTLPR | .554 | .044 | .467 | .641 | .224 |
| HTTLPR + *HTR2A* | .555 | .045 | .467 | .642 | .218 |
| HTTLPR + rs2254298 | .566 | .044 | .480 | .652 | .138 |
| HTTLPR + rs2268494 | .546 | .044 | .460 | .633 | .300 |
| HTTLPR + rs2301261 | .541 | .044 | .454 | .628 | .358 |
| HTTLPR + rs53576 | .572 | .044 | .485 | .659 | .106 |
| HTTLPR + *HTR2A* + rs2254298 | .579 | .044 | .493 | .666 | .076 |
| HTTLPR + *HTR2A* + rs2268494; | .546 | .044 | .459 | .633 | .305 |
| HTTLPR + *HTR2A* + rs2301261 | .550 | .044 | .463 | .637 | .264 |
| HTTLPR + *HTR2A* + rs53576 | .568 | .044 | .481 | .655 | .127 |
| HTTLPR + rs2254298 + rs2268494 | .555 | .044 | .469 | .642 | .215 |
| HTTLPR + rs2254298 + rs2301261 | .531 | .044 | .445 | .618 | .480 |
| HTTLPR + rs2254298 + rs53576 | .586 | .044 | .500 | .671 | .054 |
| HTTLPR + rs2268494 + rs2301261 | .535 | .044 | .449 | .622 | .429 |
| HTTLPR + rs2268494 + rs53576 | .561 | .044 | .475 | .647 | .170 |
| HTTLPR + rs2301261 + rs53576 | .565 | .044 | .479 | .651 | .145 |
| HTTLPR + *HTR2A* + rs2254298 + rs2268494; | .567 | .044 | .480 | .653 | .135 |
| HTTLPR + *HTR2A* + rs2254298 + rs2301261 | .558 | .044 | .471 | .644 | .195 |
| **HTTLPR + *HTR2A* + rs2254298 + rs53576** | **.595** | **.044** | **.509** | **.681** | **.033** |
| HTTLPR +*HTR2A* + rs2268494 + rs2301261 | .546 | .044 | .459 | .632 | .303 |
| HTTLPR + *HTR2A* + rs2268494 + rs53576 | .560 | .044 | .473 | .647 | .176 |
| HTTLPR + *HTR2A* + rs2301261 + rs53576 | .567 | .044 | .480 | .653 | .135 |
| HTTLPR + rs2268494 + rs2301261 + rs53576 | .555 | .044 | .468 | .641 | .221 |
| HTTLPR + rs2254298 + rs2268494 + rs2301261 | .528 | .044 | .441 | .614 | .532 |
| HTTLPR + *HTR2A* + rs2254298 + rs2268494 + rs2301261 | .551 | .044 | .465 | .638 | .250 |
| HTTLPR + *HTR2A* + rs2254298 + rs2268494 + rs53576 | .581 | .044 | .495 | .667 | .069 |
| HTTLPR + *HTR2A* + rs2254298 + rs2301261 + rs53576 | .577 | .044 | .491 | .663 | .085 |
| HTTLPR + *HTR2A* + rs2268494 + rs2301261 + rs53576 | .562 | .044 | .475 | .648 | .166 |
| HTTLPR + *HTR2A* + rs2254298 + rs2268494 + rs2301261 + rs53576 | .567 | .044 | .481 | .653 | .134 |
| *HTR2A* | .530 | .045 | .443 | .618 | .494 |
| *HTR2A* + rs2254298 | .560 | .044 | .473 | .647 | .179 |
| *HTR2A* + rs2268494 | .523 | .044 | .437 | .610 | .598 |
| *HTR2A* + rs2301261 | .533 | .045 | .445 | .620 | .461 |
| *HTR2A* + rs53576 | .547 | .044 | .460 | .634 | .293 |
| *HTR2A* + rs2254298 + rs2268494 | .549 | .044 | .462 | .635 | .274 |
| *HTR2A* + rs2254298 + rs2301261 | .537 | .045 | .450 | .624 | .408 |
| *HTR2A* + rs2254298 + rs53576 | .572 | .044 | .486 | .659 | .105 |
| *HTR2A* + rs2268494 + rs2301261 | .532 | .044 | .445 | .618 | .478 |
| *HTR2A* + rs2268494 + rs53576 | .539 | .044 | .452 | .626 | .385 |
| *HTR2A* + rs2301261 + rs53576 | .546 | .044 | .459 | .633 | .301 |
| *HTR2A* + rs2254298 + rs2268494 + rs2301261 | .536 | .044 | .449 | .622 | .425 |
| *HTR2A* + rs2254298 + rs2268494 + rs53576 | .560 | .044 | .474 | .647 | .175 |
| *HTR2A* + rs2254298 + rs2301261 + rs53576 | .552 | .044 | .466 | .639 | .239 |
| *HTR2A* + rs2268494 + rs2301261 + rs53576 | .545 | .044 | .459 | .632 | .307 |
| *HTR2A* + rs2254298 + rs2268494 + rs2301261 + rs53576 | .549 | .044 | .462 | .635 | .276 |
| rs2254298 | .532 | .044 | .445 | .618 | .478 |
| rs2254298 + rs2268494 | .527 | .044 | .441 | .614 | .540 |
| rs2254298 + rs2301261 | .507 | .044 | .420 | .594 | .879 |
| rs2254298 + rs53576 | .558 | .044 | .472 | .644 | .194 |
| rs2254298 + rs2268494 + rs2301261 | .507 | .044 | .421 | .594 | .867 |
| rs2254298 + rs2268494 + rs53576 | .546 | .044 | .459 | .632 | .305 |
| rs2254298 + rs2301261 + rs53576 | .531 | .044 | .444 | .618 | .486 |
| rs2254298 + rs2268494 + rs2301261 + rs53576 | .527 | .044 | .441 | .614 | .540 |
| rs2268494 | .501 | .045 | .414 | .589 | .976 |
| rs2268494 + rs2301261 | .507 | .044 | .420 | .594 | .883 |
| rs2268494 + rs53576 | .537 | .044 | .450 | .624 | .407 |
| rs2268494 + rs2301261 + rs53576 | .535 | .044 | .448 | .622 | .436 |
| rs2301261 | .507 | .045 | .419 | .594 | .883 |
| rs2301261 + rs53576 | .540 | .044 | .452 | .627 | .374 |
| rs53576 | .543 | .044 | .457 | .630 | .329 |

^1^ Null hypotheses: area = 0.5.

Abbreviations: ASD, autism spectrum disorder; AUC, area under the curve; CI, asymptotic 95% confidence interval; HTR2A, serotonin receptor 2A; HTTLPR, serotonin-transporter–linked polymorphic region; OXTR, oxytocin receptor; SD, standard deviation; **Bold**, nominal significant.
